# The effects of backward vs. forward running training on measures of physical fitness in young female handball players

**DOI:** 10.3389/fspor.2023.1244369

**Published:** 2023-09-12

**Authors:** Senda Sammoud, Raja Bouguezzi, Aaron Uthoff, Rodrigo Ramirez-Campillo, Jason Moran, Yassine Negra, Younes Hachana, Helmi Chaabene

**Affiliations:** ^1^ Research Laboratory (LR23JS01) «Sport Performance, Health & Society», Higher Institute of Sport and Physical Education of Ksar Saïd, University of “La Manouba”, La Manouba, Tunisia; ^2^ High Institute of Sports and Physical Education, Kef, University of Jendouba, Jendouba, Tunisia; ^3^ Sports Performance Research Institute New Zealand (SPRINZ), AUT Millennium, School of Sport and Recreation, AUT University, Auckland, New Zealand; ^4^ Exercise and Rehabilitation Sciences Laboratory, School of Physical Therapy, Faculty of Rehabilitation Sciences, Universidad Andres Bello, Santiago, Chile; ^5^ School of Sport, Rehabilitation and Exercise Sciences, University of Essex, Colchester, United Kingdom; ^6^ Higher Institute of Sport and Physical Education of Ksar Saïd, University of “La Manouba”, Manouba, Tunisia; ^7^ Department of Sports and Health Sciences, Faculty of Human Sciences, University of Potsdam, Potsdam, Germany

**Keywords:** team sports, athletic performance, motor activity, youth sports, sports medicine

## Abstract

**Introduction:**

This study examined the effects of an 8-week backward running (BR) vs. forward running (FR) training programmes on measures of physical fitness in young female handball players.

**Methods:**

Twenty-nine players participated in this study. Participants were randomly assigned to a FR training group, BR training group, and a control group.

**Results and discussion:**

Within-group analysis indicated significant, small-to-large improvements in all performance tests (effect size [*g*] = 0.36 to 1.80), except 5-m forward sprint-time in the BR group and 5- and 10-m forward sprint-time in the FR group. However, the CG significantly decreased forward sprint performance over 10-m and 20-m (*g* = 0.28 to 0.50) with no changes in the other fitness parameters. No significant differences in the amount of change scores between the BR and FR groups were noted. Both training interventions have led to similar improvements in measures of muscle power, change of direction (CoD) speed, sprint speed either forward or backward, and repeated sprint ability (RSA) in young female handball players, though BR training may have a small advantage over FR training for 10-m forward sprint time and CoD speed, while FR training may provide small improvements over BR training for RSA_best_. Practitioners are advised to consider either FR or BR training to improve various measures of physical fitness in young female handball players.

## Introduction

Competitive handball match play is characterized by high-intensity activity patterns (1). In female handball, key physical fitness components such as linear sprint, change of direction (CoD) speed, jumping ability, and repeated sprint ability (RSA) are crucial for competitive success (2). These physical attributes are crucial for handball players to excel in the dynamic nature of the game. Jumping allows for powerful shots, effective defensive blocks, and successful aerial duels (2, 3). CoD speed enables quick and agile movements to evade opponents and swiftly change attacking or defensive strategies (2). Linear sprint facilitates fast counterattacks and the pursuit of opponents (2). Finally, repeated sprint ability ensures sustained high-intensity performance throughout the game, allowing players to execute multiple high-intensity actions consecutively (3). Mastering these skills is essential for female handball players aiming to dominate the court. There is evidence that compared to lower-level competitive female handball players, higher-level ones displayed better performance in sprint over short distances, RSA, CoD speed, and horizontal jumping (4). Accordingly, the development of female handball players’ key components of physical fitness should be prioritized and optimized through well-designed conditioning programmes.

The principle of training specificity dictates that the greatest improvements will occur in the athletic task that presents similar features from biomechanical and physiological standpoints with the trained exercise (5–7). However, there are indications that changes could also occur in movements that were not specifically trained (7, 8). For example, Negra et al. (8) revealed moderate improvement in RSA parameters (i.e., RSA_total time_, and RSA_best time_) following either nine weeks of specific (i.e., RSA with CoD) or unspecific (i.e., RSA without CoD) training interventions in youth male soccer players aged 15 years. As per specific training methods, recent evidence indicated that non-specific training such as reverse-movement training [i.e., backward running (BR)] may also have the potential to stimulate positive adaptations with marked transfer to athletic tasks (9). Indeed, several studies (8–10) supported the utility of BR training to improve a range of physical fitness components related to both maximal neuromuscular performance (e.g., sprint, CoD, and jumping ability) and cardiorespiratory functioning (e.g., running economy). For instance, Terbalanche et al. (10) suggested that netball-specific exercises performed backwards, can be included in the conditioning and skills training programmes to improve speed, CoD (i.e., 505 CoD, Ladder, and *T*-test), and power measures. Likewise, Ordway et al. (11) noted improvement in forward running (FR) economy (2.54%) without altering maximal oxygen consumption (VO_2max_) or body composition after ten-week of BR training in trained young male runners aged 26 years. Further, Uthoff et al. (9) showed greater positive effects on 10 m forward sprint speed [effect size (ES) = −0.47], 20-m forward sprint speed (ES = −0.26), and countermovement jump (CMJ) height (ES = 0.51) after eight-week of biweekly BR training compared to FR training in adolescent male athletes aged 15 years.

Overall, based on the existing literature, it seems that BR training leads to positive effects on various measures of physical fitness (10, 12) which could even exceed those achieved following FR training (12). However, to the best of our knowledge, there is no study available that directly contrasted the effects of FR vs. BR training on key measures of physical fitness in young female handball players, pointing to a gap in the literature. Therefore, this study aimed to examine the effects of an eight-week in-season BR training vs. FR training, in combination with regular handball training, on key measures of physical fitness in young female handball players. We hypothesized, that BR training would result in greater enhancements in measures of physical fitness compared to FR training (12).

## Methods

### Experimental design

A three-group randomized-controlled trial design was applied to examine the effects of FR training vs. BR training on measures of physical fitness in young female handball players. For two sessions per week, the first part of the regular handball training of the participants was replaced with either FR training or BR training. The control group (CG) continued to undertake its regular handball training. The pre-and post-intervention assessments included tests of forward and backward sprint speed (i.e., 20 m with 5- and 10 m split intervals), forward CoD speed (i.e., 505 CoD test), jumping ability [i.e., standing long jump (SLJ)], and forward RSA. All tests were conducted 48 h after the players' most recent training session or match, at the same time of day (7:30–9:30 a.m.) and under the same environmental conditions (29–33°C, no wind). Players who failed to attend 85% of the scheduled training sessions were excluded from the study. Physical fitness tests were performed in a fixed order over two days. On the first day, anthropometric measurements were conducted, followed by the sprint speed and the CoD speed tests. The second day was devoted to the jumping and RSA tests. Two experienced conditioning trainers, who were blinded to group allocation, conducted all measurements.

### Participants

We conducted an *a priori* sample size calculation for the 10 m sprint test. We set *α* at 0.05 and the statistical power at 0.80. The estimated effect size of Cohen's *d* = 1.20 is based on a similar study (9). Therefore, the required number of participants per group was determined to be six. To account for potential participant attrition twenty-nine female handball players, from the same regional handball team, were recruited to take part in the study. Of note, all the groups followed the same handball training program under the supervision of the same coaches. Participants were randomly assigned to a FR training group (*n* = 9), a BR training group (*n* = 9), or an active CG (n = 11). The anthropometric characteristics of the three groups are detailed in Table 1. All participants were classified as experienced handball players with 9.0 ± 1.3 years of systematic handball training experience involving four-to-five training sessions per week. All players met the following inclusion criteria: (i) they had undertaken continuous handball training over the past three months with no musculoskeletal injuries sustained, (ii) absence of potential medical problems that could compromise participation in the study, and (iii) none were engaged in any other sport or played with any other handball club.

**Table 1:** Participants’ characteristics (mean ± standard deviation).

Parameters	Overall (n = 29)	BR group (n = 9)	FR group (n = 9)	Control group (n = 11)
Age (y)	20.1 ± 2.17	20.1 ± 2.15	19.9 ± 2.62	20.2 ± 1.99
Body height (cm)	1.67 ± 0.07	1.66 ± 0.05	1.70 ± 0.66	1.64 ± 0.09
Body mass (kg)	64.2 ± 9.93	61.3 ± 7.57	70.0 ± 10.3	61.9 ± 10.1

BR, backward running; FR, forward running.

The study was conducted as per the latest Declaration of Helsinki, and the protocol was approved by the local Ethics Committee of the «blind for review purposes». Signed written informed consent (parents/legal guardians) and assent (participants) were obtained before the commencement of the study. All participants were told that they were free to withdraw from the study without any penalty at any time, with no obligation to explain.

### Training programmes

The programmes were conducted during the second half of the in-season period. All groups participated in the same regular handball-specific training program over the eight-week intervention period. Handball training sessions for all groups included fast footwork drills, technical skills and moves, position games, and tactical games. The FR and BR training sessions were integrated into the regular handball training routine of the intervention groups after their standard warm-up, replacing 20–25 min of low-intensity handball drills, on Tuesdays and Thursdays (Table 2). After either the FR or BR training sessions, the players completed the remainder (60–70 min) of their regular handball training. The running training program involved players performing linear running either forward or backward. The players performed slow, moderate, and fast sprints, corresponding to ∼20–45, ∼50–75, and >95% of maximal sprint effort, respectively. These speeds were chosen to reflect common running intensities that young female players are capable of self-selecting using autoregulation. Similar to a previous study (9), coaching cues were used to reinforce the BR technique (i.e., “slight lean of the chest forward”, “use similar arm action to FR”, and “high heel recovery of the swing leg”). Likewise, specific technical instructions, such as; (a) “knee-up and toe-up,” (b) “drive your arms from cheek to hip,” (c) “strike the ground with the ball of your foot,” and (d) “strike the ground under your hips and push back” were used to reinforce FR techniques. The repetitions by intensity over the prescribed distances for each training session were detailed in Table 2. Equal volume and intensity were prescribed for both the FR and BR training groups.

**Table 2:** Forward and backward running intervention programs.

		Intensity	Sets (n)	Distance (m)	Distance per intensity (m)	Total distance (m)
Week 1	Session 1	Slow	3	15	45	225
		Moderate	4	15	60	
		Fast	8	15	120	
	Session 2	Slow	3	15	45	225
		Moderate	3	15	45	
		Fast	9	15	135	
Week 2	Session 1	Slow	2	15	30	225
		Moderate	4	15	60	
		Fast	9	15	135	
	Session 2	Slow	2	15	30	225
		Moderate	3	15	45	
		Fast	10	15	150	
Week 3	Session 1	Slow	1	15	15	225
		Moderate	4	15	60	
		Fast	10	15	150	
	Session 2	Slow	2	15	30	225
		Moderate	2	15	30	
		Fast	11	15	165	
Week 4	Session 1	Slow	1	15	15	225
		Moderate	3	15	45	
		Fast	11	15	165	
	Session 2	Slow	1	15	15	225
		Moderate	2	15	30	
		Fast	12	15	180	
Week 5	Session 1	Slow	3	20	60	300
		Moderate	4	20	80	
		Fast	6	20	160	
	Session 2	Slow	3	20	60	300
		Moderate	3	20	60	
		Fast	9	20	180	
Week 6	Session 1	Slow	2	20	40	300
		Moderate	4	20	80	
		Fast	9	20	180	
	Session 2	Slow	2	20	40	300
		Moderate	3	20	60	
		Fast	10	20	200	
Week 7	Session 1	Slow	1	20	20	300
		Moderate	4	20	80	
		Fast	10	20	200	
	Session 2	Slow	2	20	40	300
		Moderate	2	20	40	
		Fast	11	20	220	
Week 8	Session 1	Slow	1	20	20	300
		Moderate	3	20	60	
		Fast	11	20	220	
	Session 2	Slow	1	20	20	300
		Moderate	2	20	40	
		Fast	12	20	240	

### Forward and backward linear sprint speed time

Twenty-meter forward and backward linear sprint speed time was assessed across 20 m distance with split intervals at 5- and 10 m using a single beam electronic timing system (Microgate SRL, Bolzano, Italy). Participants started in a standing split stance position with their lead foot 0.3 m behind the first infrared photoelectric gate, which was placed 0.75 m above the ground to ensure that it captured trunk movement and avoided false signals through limb motion. In total, four single-beam photoelectric gates were used. No rocking or false steps were permitted before starting. The between-trial recovery time was 3 min. The best performance out of three trials was used for further analysis. The intra-class correlation coefficients (ICCs) for test-retest reliability were 0.91, 0.93, and 0.90 for 5-, 10- and 20 m forward sprint performance, respectively, and 0.87, 0.82, and 0.85 for 5-, 10- and 20 m backward sprint performance, respectively.

### 505 change of direction speed test

The 505 CoD speed test was administered as previously outlined by Negra et al. (8) using an electronic timing system (Microgate, Bolzano, Italy) placed 5 m from the turning line. Players assumed a standing split stance position 10 m from the start line, ran as quickly as possible through the start/finish line, pivoted 180° on their preferred leg at the 15 m turning line indicated by a cone marker, and returned as fast as possible through the start/finish line. To ensure proper execution of the test, a researcher was positioned at the turning line and if the participant changed direction before reaching the turning point, or turned off the incorrect foot, the trial was disregarded and reattempted after 3 min recovery period. A between-trial rest period of 3 min was provided. The best performance out of three trials was used for further analysis. The ICC for test-retest reliability was 0.91.

### Standing-long-jump distance

During the bilateral SLJ test, participants stood with their feet shoulder-width apart and their toes behind a starting line. Participants performed a fast flexion of the legs and downward movement of the arms, before jumping forward as far as possible. Participants had to land with both feet at the same time and were not allowed to fall forward or backward. The horizontal distance between the starting line and the heel of the rear foot was recorded using a tape measure to the nearest 1-cm. A between-trial rest period of 1 min was allowed. The best out of three trials was recorded for further analysis. The ICC for test-retest reliability was 0.87.

### Repeated sprint ability

The RSA test was assessed via the same photocell system used for the linear speed and 505 CoD tests (Microgate, Bolzano, Italy). Immediately after the standardized warm-up, participants completed a preliminary single shuttle-sprint test (20 + 20 m with 180° CoD). The first trial provided the criterion score for the actual shuttle-sprint test (13). Participants then rested for 5 min before starting the RSA test. During the first sprint, participants had to achieve at least 97.5% of their criterion score, otherwise, they rested for 5 min and then restarted the test (13). We used such an approach to determine if participants adopted a coping strategy for performance. Of note, all participants attained their criterion score during the first sprint. All participants performed six 20 m shuttle sprints with 180° turns, separated by 25 s of passive recovery (13). Three seconds prior to the commencement of each sprint, players were asked to adopt the ready position using a split stance with their lead foot 0.3 m behind the starting line until the next start signal. From the starting line, they sprinted for 20 m, touched the second line with one foot, performed a 180° CoD, and returned to the starting line as quickly as possible. Participants were instructed to complete all sprints as fast as possible. The RSA best time (RSA_best_), and the RSA total time (RSA_tot_) were recorded. Due to the fatigue induced by the test, only one maximal attempt was made i.e., no ICC was calculated. The reliability of this test was examined elsewhere (14).

### Statistical analyses

The statistical analyses were conducted using Microsoft Excel (version 16.0; Microsoft Corporation, Seattle, WA, USA) and SPSS 28.0 for Windows (SPSS, IBM Corporation, Chicago, IL, USA). The data were explored using histogram plots and distribution estimation, and the normality of the distribution for all variables was tested and confirmed using the Shapiro-Wilk test. Levene's test was used to assess the homogeneity of variance. Taking a frequentist approach, a repeated measured ANOVA was used to compare within-group pre- post-training performance, helping minimize false positives which can arise during multiple comparisons. Between-group training-related effects were assessed using a one-way analysis of variance (ANOVA) on the change scores (mean differences from pre-training to post-training) (9). Sidak posthoc corrections were applied to locate pairwise differences between groups. Alpha was set at *p* < 0.05% and 95% confidence intervals (CI) were used for all analyses. To quantify the magnitude of the performance change both within- and between-group, percentage change and Hedges *g* effect size statistics were calculated (15), with ES magnitudes of <0.2, ≥0.2–0.49, ≥0.5–0.79, and >0.8 classified as trivial, small, moderate, and large, respectively (16, 17). To determine the practical relevance of performance changes, the smallest worthwhile individual change (SWC) was calculated on the pooled standard deviation (SD) of pre-training session scores for all groups and converted to a percentage for each performance variable, where changes were deemed small (SWC = 0.2 × SD), moderate (MWC = 0.6 × SD), or large (LWC = 1.2 × SD) (9, 15).

## Results

No injuries were reported as part of the training programmes. Within-group changes from pre- to post-training and between-group comparisons are presented in Table 3. The within-group analysis revealed that the BR training induced significant improvements in most performance tests, except forward and backward sprinting over 5 m, forward sprinting over 20 m, and RSA_best_ (*p *< 0.05; Δ4.37%–11.0%; *g *= 0.46–1.80). The FR training induced significant within-group improvements for 10 m and 20 m backwards sprints, 505 CoD, and SLJ (*p *< 0.05; 5.57%–8.65%; *g *= 0.36–1.29). Meanwhile, no significant changes were observed in any of the fitness parameters for the CG.

**Table 3:** Descriptive performance testing results with for BRT, FRT, and CG groups including within-group changes from pre- to post-training and between-group differences of the mean changes.

Performance Test	Group	Pre (mean ± SD)	Post (mean ± SD)	Performance change (%) (95% CI)	Post-pre training effect (ES)	Diff BRT-CG (mean ± SE)	Effect size	Diff FRT-CG (mean ± SE)	Effect size	Diff BRT-FRT (mean ± SE)	Effect size
Backward 5 m sprint (s)	BR	1.57 ± 0.29	1.41 ± 0.09	−8.30 (−16.4 to −0.21)	−0.58	−0.13 ± 0.09	−0.55**^B^**	−0.11 ± 0.06	−0.62**^F^**	−0.02 ± 0.10	−0.08**^B^**
	FR	1.64 ± 0.19	1.50 ± 0.04	−7.46 (−13.3 to −1.64)	−0.78						
	CG	1.65 ± 0.12	1.61 ± 0.11	−1.77 (−3.30 to −0.25)	−0.21						
Backward 10 m sprint (s)	BR	2.84 ± 0.51	2.50 ± 0.15*	−10.3 (−17.7 to −2.92)	−0.71	−0.18 ± 0.15	−0.43**^B^**	−0.12 ± 0.09	−0.40**^F^**	−0.06 ± 0.27	−0.13**^B^**
	FR	2.99 ± 0.33	2.71 ± 0.12*	−8.65 (−13.2 to −4.09)	−0.88						
	CG	3.05 ± 0.21	2.89 ± 0.19	−5.18 (−7.20 to −3.16)	−0.67						
Backward 20 m sprint (s)	BR	5.17 ± 0.94	4.53 ± 0.32**✧**	−10.9 (−17.6 to −4.15)	−0.71	−0.26 ± 0.25	−0.33**^B^**	−0.15 ± 0.14	−0.29**^F^**	−0.10 ± 0.27	−0.12**^B^**
	FR	5.44 ± 0.55	4.90 ± 0.31*	−8.48 (−13.0 to −6.00)	−0.94						
	CG	5.96 ± 0.42	5.48 ± 0.37	−6.40 (−8.75 to −4.05)	−0.80						
Forward 5 m sprint (s)	BR	1.09 ± 0.06	1.07 ± 0.06	−1.35 (−3.80 to 1.11)	−0.22	−0.05 ± 0.02	−0.78**^B^**	−0.05 ± 0.04	−0.63**^F^**	−0.01 ± 0.03	−0.10**^B^**
	FR	1.16 ± 0.07	1.15 ± 0.11	−0.71 (−5.88 to 4.45)	−0.07						
	CG	1.13 ± 0.07	1.17 ± 0.06	3.45 (0.83 to 6.24)	0.50						
Forward 10 m sprint (s)	BR	1.92 ± 0.07	1.87 ± 0.11*	−2.70 (−4.28 to −1.12)	−0.45	−0.11 ± 0.03	−0.73**^B^**	−0.06 ± 0.05	−0.37**^F^**	−0.04 ± 0.05	−0.38**^B^**
	FR	2.05 ± 0.13	2.05 ± 0.17	−0.20 (−4.89 to 4.48)	−0.03						
	CG	2.10 ± 0.17	2.15 ± 0.16	2.69 (0.59 to 4.79)	0.28						
Forward 20 m sprint (s)	BR	3.41 ± 0.18	3.35 ± 0.15	−1.71 (−3.54 to 0.11)	−0.29	−0.06 ± 0.05	−0.29**^B^**	−0.10 ± 0.06	−0.41**^F^**	0.04 ± 0.06	0.16**^F^**
	FR	3.75 ± 0.24	3.66 ± 0.21	−2.51 (−6.11 to 0.08)	−0.35						
	CG	3.83 ± 0.20	3.84 ± 0.21	0.09 (01.79 to 1.96)	0.01						
505 COD (s)	BR	2.93 ± 0.12	2.61 ± 0.16**†**	−11.0 (−14.2 to −7.88)	−1.80	−0.31 ± 0.06**†**	−1.74**^B^**	−0.24 ± 0.07**✧**	−1.11**^F^**	−0.06 ± 0.08	−0.41**^B^**
	FR	3.05 ± 0.14	2.79 ± 0.18**†**	−8.58 (−12.2 to −4.95)	−1.29						
	CG	3.19 ± 0.08	3.17 ± 0.15	−0.62 (−2.60 to 1.36)	−0.13						
Standing long jump (m)	BR	1.81 ± 0.14	1.89 ± 0.13*	4.37 (2.33 to 6.42)	0.46	0.10 ± 0.02**✧**	0.55**^B^**	0.11 ± 0.03**✧**	0.55**^F^**	−0.01 ± 0.03	−0.05**^F^**
	FR	1.55 ± 0.17	1.64 ± 0.21**✧**	5.57 (1.72 to 9.42)	0.36						
	CG	1.42 ± 0.18	1.40 ± 0.18	−1.29 (−3.51 to 0.94)	−0.08						
RSA_best_ (s)	BR	8.19 ± 0.35	8.00 ± 0.38	−2.27 (−4.22 to −0.31)	−0.40	−0.18 ± 0.14	−0.44**^B^**	−0.33 ± 0.19	−0.70**^F^**	0.15 ± 0.17	0.32**^F^**
	FR	8.92 ± 0.48	8.58 ± 0.53	−3.72 (−6.98 to −0.46)	−0.53						
	CG	9.44 ± 0.38	9.43 ± 0.38	−0.04 (−2.33 to 2.25)	−0.02						
RSA_total_ (s)	BR	52.0 ± 2.53	49.5 ± 2.08*	−4.82 (−7.04 to −2.61)	−0.86	−2.42 ± 0.96	−0.83^B^	−1.87 ± 1.24	−0.64**^F^**	−0.55 ± 1.16	−0.19**^B^**
	FR	55.5 ± 2.59	53.5 ± 3.61	−3.57 (−7.04 to −0.11)	−0.50						
	CG	58.6 ± 2.71	58.4 ± 2.20	−0.12 (−2.59 to 2.35)	−0.05						

BR, backward running training; FR, forward running training; CG, control group; COD, Change of direction; RSA, repeat sprint ability; M, mean; SD, standard deviation; ES, effect size; SE, standard error; CI, confidence interval. *,✧,†, significant for within-group and between-group comparisons (*p* < 0.050, 0.010, and 0.001, respectively); B, training effect favouring BRT; F, training effect favouring FRT.

The one-way ANOVA on the change scores showed a significant group effect for the 505 CoD (*F* = 13.204; *p* < 0.001) and SLJ (*F* = 8.654; *p* = 0.001). The posthoc analysis indicated that, compared with the CG, the change scores for the BR and FR groups were significantly better for 505 CoD time and SLJ (*g *= 0.55–1.74). There were no significant differences in the amount of change scores from pre- to post-test between the BR and FR groups.

**Figure 1:**
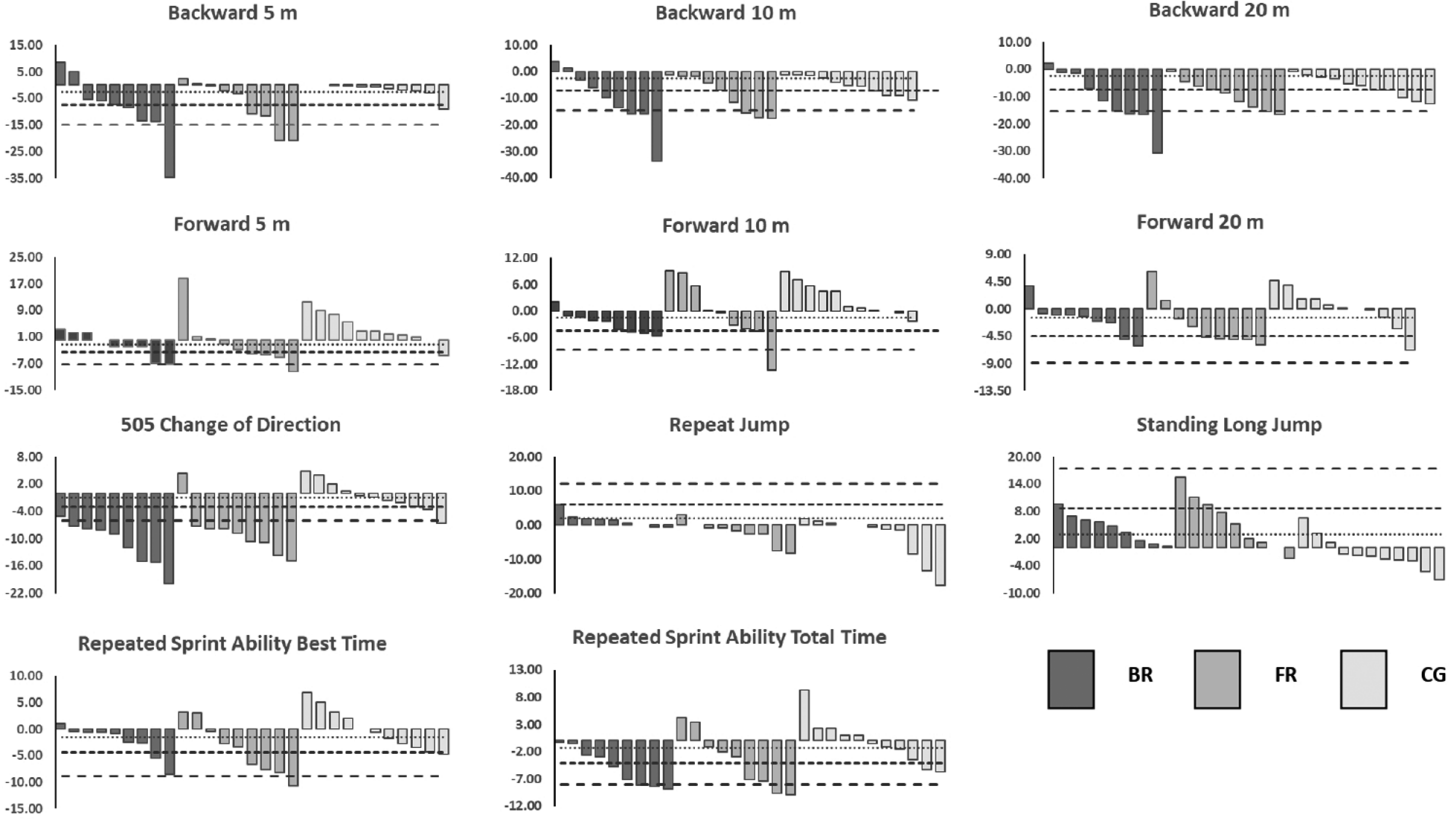
Individual percentage change from pre-training to post-training for performance tests relative to small, moderate, and large worthwhile change in performance. ………… Small response (SWC = 0.2); ………… Moderate response (MWC = 0.6); _ _ _ _ _ _ . Large response (LWC = 1.2); FRT, forward running training group; BRT Backward running training group; CON, control group.

Figure 1 provides a graphical reference illustrating the individual percentage changes relative to the magnitudes of worthwhile change detected for the BR, FR, and CG groups for the different performance tests. The BR and FR groups achieved similar individual response rates across all performance tests, with an average of 6, 3, and 2 participants improving above the SWC, MWC, and LWC in the BR group and 5, 4, and 2 participants improving beyond these thresholds in the FR group, respectively. Meanwhile, only 3, 1, and 0 participants from the CG improved beyond the SWC, MWC and LWC, respectively, across the different performance tests. All participants in the BR group improved 505 CoD performance above the MWC, while 8 out of the 9 participants met the threshold for a LWC. The FR and BR groups had each 8 of 9 athletes above the SWC and LWC for the 505 CoD test. Regarding the CG, two or fewer subjects improved performance above the SWC in the performance tests, and while no significant changes were found, 9 out of the 11 participants improved backward 20 m sprint times above the SWC threshold.

## Discussion

This study aimed to examine and compare the effects of eight weeks of BR training vs. FR training on measures of physical fitness in female handball players. The main findings indicated that the vast majority of measures of physical fitness improved after both training interventions. More specifically, significant small to large improvements in all performance tests, except 5- and 20 m forward sprint-time, were observed in the BR group. Meanwhile, significant small to large enhancements in 10 m and 20 m backwards sprints, 505 CoD, and SLJ were noted for the FR group. Additionally, the between-group analysis indicated no significant differences in the amount of change scores between the two training interventions.

To the authors' knowledge, this is the first study to directly contrast the effects of BR vs. FR training on measures of physical fitness in young female handball players. Additionally, this study seems to be unique to integrate backward running performance in its testing battery. Although only significant over 10 m and 20 m, BR training improved backward running performance to a moderate effect (*g* = 0.58–0.71) and FR training led to moderate to large improvements in backward running performance (*g* = 0.78–0.94), with both groups showing small to moderate effectiveness, though not statistically significant compared to the CG (*g* = 0.29–0.62). In terms of individual response rates, over half of the participants of both BR and FR groups improved their performances beyond the SWC and nearly reached the threshold for MWC. Interestingly, just over half of the participants of the CG also improved average backward running performance above the SWC threshold. While neither intervention group statistically improved performance relative to the CG, average change score improvements for the BR group were 0.16–0.39 s faster than the CG and average change scores for the FR group were 0.06–0.12 s better than the CG across all distances. These findings suggest that BR and FR training are better than handball training alone and that while either locomotive direction of training could be used to improve backward running performance, BR appears to induce the most favorable responses. Since handball players have been found to spend an average of 1.4%–2.92% of total playing time running backwards (3, 18), with wings showing the greatest total distance covered using this locomotive technique (3), enhancing this physical trait may transfer to better on-court performance. However, since this is the first study to include BR in an empirically scrutinized testing battery, more research is required to understand the trainability of this direction of running and its actual transference to on-court capabilities.

High levels of linear speed over short and medium (<30 m) distances are important physical fitness attributes in female handball players (2). BR training has been previously found to improve forward sprint performance in youth athletes (9) and maintain this athletic quality relatively better than FR training in well-trained female netball athletes (10). A similar trend was oberserved in the current study, where only BR training was found to significantly enhance forward 10 m sprint performance. BR training led to small improvements across all sprinting distances (Δ1.35%–2.70%; *g* = 0.22–0.45), the FR training induced trivial changes to 10 m and 20 m distances (*Δ*0.71%–2.51%; *g* = 0.03–0.35), and both groups led to small to moderate enhancements compared to the CG across all distances (*g* = 0.29–0.78). The relative response rates were similar for both BR training and FR training, with over half of the participants in each group improving performance beyond the SWC. These relative response rates are lower than previously reported for BR training (∼96%) but similar to responses following FR training (9). While the within-group changes following BR training are in line with the 2.54% increase in performance over 5 m–20 m forward sprints in ∼20 year-old netball athletes (10), they are much lower than the 6.37% average performance increase over 10 m and 20 m distances observed in 14.6 year-old male athletes (9). Given that youth and adult athletes respond differently to exercise (19), the novel stimuli associated with BR could explain the discrepancies observed between those of Uthoff et al. (9) and the current study. Nonetheless, both FR and BR can be used to enhance forward linear sprint ability up to 20 m, though, the findings in this study substantiate previous observations that BR appears to preferentially enhance shorter sprint performance (i.e., ≤10 m) whereas FR leads to more pronounced improvements over longer sprint distances (i.e., ≥20 m) (9).

The ability to change directions effectively is a distinctive feature of success in female handball players (), spending ∼6.92% of their time during matches performing this athletic task (3). Therefore, developing CoD ability of a handball player is likely to be advantageous in on-court competition (2). Our findings showed large pre-to-post improvements in both experimental groups (*g *≥ 1.29) and large positive effects compared to the CG group (*g *≥ 1.11). A higher relative number of participants in the BR and FR groups experienced adaptations to CoD performance greater than the SWC compared with the CG, with all participants in the BR group and all but one participant in the FR group experienced improvements beyond the MWC. Albeit not significant, the BR training induced a small improvement relative to the FR training. The 11.0% and 8.58% increases in 505 CoD performance associated with BR training and FR training, respectively, are greater than the relative 3.18% and 0.75% changes observed by Terblanche and Venter (10) in netball athletes following 6-weeks of netball-specific BR or FR training, respectively. The growing empirical support suggests that BR training and FR training can both be used to improve 505 CoD performance, though BR training appears to have a small advantage compared to FR training. Importantly, however, since the 505 CoD test comprises multiple phases (20, 21), it is unclear whether BR training or FR training leads to phase-specific adaptations (i.e., deceleration, directional change, or reacceleration). Research in this area may help elucidate the effects of running direction on phase-specific adaptations during CoD tasks.

Regarding SLJ, the current study revealed that both BR training and FR training resulted in small, yet significant improvements in SLJ (Δ4.37%–5.57%). Additionally, both interventions were found to be moderately more effective than the CG (*g *= 0.55). Individual analysis indicated that six out of nine in the BR group, and five out of nine in the FR group displayed improvements above the SWC, while only two out of eleven achieved this level of improvement in the CG. These outcomes contradict our expectations that vector-specific training to the SLJ (i.e., FR training) would lead to better improvemtents compared to a non-vector-specific program (i.e., BR training). Indeed, BR training was found to be similarly effective for enhancing horizontal slow-stretch shortening cycle jumping ability. Furthermore, given the dominance of the contractile tissue during BR training (12, 22), squat jump performance may be more sensitive to the stimulus associated with BR because the pause at the end of the eccentric phase will result in less contribution from the elastic tissues on the subsequent concentric phase of the jump (23). Future research should examine the influence of BR training vs. FR training on jump types using differing degrees of elastic utilization.

The ability to repeatedly produce maximal sprint efforts with minimal recovery time is a key physical component for highly-trained female handball players due to the intermittent activity nature that characterize match play and training (3). It has been shown that RSA performance can effectively differentiate between professional- and amateur-level female handball players (18). The current results found that FR training induced moderate within-group improvements for both RSA_best_ (3.72%) and RSA_total_ (3.57%) performance, while BR training led to a small improvement in RSA_best_ (2.27%) and large improvement for RSA_total_ (4.82%) performance, with only the changes to RSA_total_ in the BR group achieving statistical significance. These small to large improvements are greater than the 1.68% and 1.62% improvements for best and average RSA, respectively, following ten weeks of complex strength training in ∼17-year-old female handball players (5), and greater than the 0.83% RSA_best_ and 2.30% RSA_total_ improvements observed in 15–16-year-old female handball players after 8-week of plyometric training (24). Though direct comparisons with previous studies are not conclusive, based on the results of the current study, BR and FR training appear to be effective methods for healthy, trained female handball players to improve the RSA better than previously used methods such as complex strength training or plyometric training. A high inverse correlation has been found between RSA ability and maximum oxygen consumption in handball players (25). Meaning that players with greater aerobic capacity will demonstrate better RSA. However, while BR training has been previously shown to improve FR economy, it has not been found to alter maximum oxygen consumption (11). Given that BR training has been found to improve both RSA and FR economy without associated increases in maximum oxygen consumption, an alternative metabolic (e.g., PCr recovery and H^+^ buffering) or neuromuscular changes (e.g., neural drive and motor-unit recruitment) may be stimulating these adaptations.

In conclusion, the findings of the current study suggest that both BR and FR training can be used to improve backward and forward linear sprinting, CoD, horizontal jumping, and RSA performance in well-trained female handball players, though BR training may have a small advantage over FR training for 10 m sprint 505 CoD, and RSA_total_.

Though the effectiveness of BR training on measures of athletic performance has been explored since the mid-to-late 2000’s (10, 26), empirical evidence in this area is still limited, with only a handful of studies looking at the performance adaptations associated with this direction of running (9–11, 26, 27). Therefore, more research on this topic should be conducted to gain a better understanding of direction-specific adaptations associated with BR and FR on phase-specific CoD performance, jump types utilizing varying degrees of elastic contribution, and the physiological and neuromuscular responses underpinning aerobic adaptations. It is important to note that the present results should be further confirmed through future studies, particularly those involving longer training periods exceeding 8 weeks. Extending the duration of the training program may provide additional insights into the long-term effects and sustainability of the observed improvements.

## Conclusion

Both forward and BR training can be used, in combination with the handball training routine, to improve backward and forward sprinting, CoD, horizontal jumping, and RSA in young highly-trained female handball players. Practitioners working with youth female handball players are advised to consider implementing either FR or BR training into the training schedule. It should be mentioned though that BR training may have a small advantage over FR training for forward 10 m sprint, 505 CoD speed, while FR training may provide small improvements over BR training for RSA_best_.

## Data Availability

The raw data supporting the conclusions of this article will be made available by the authors, without undue reservation.
